# Sacral Ewing sarcoma with rib, lung, and multifocal skull metastases: A rare case report and review of treatments

**DOI:** 10.3389/fonc.2022.933579

**Published:** 2022-09-08

**Authors:** Chen Ye, Wei Wei, Xuebin Tang, Feng Li, Baoquan Xin, Qianqian Chen, Haifeng Wei, Shaohui He, Jianru Xiao

**Affiliations:** ^1^ School of Health Science and Technology, University of Shanghai for Science and Technology, Shanghai, China; ^2^ Spinal Tumor Center, Department of Orthopaedic Oncology, Changzheng Hospital, Second Military Medical University, Shanghai, China; ^3^ Department of Orthopaedics, The Second Affiliated Hospital of Xuzhou Medical University, Xuzhou, China; ^4^ Department of Orthopaedics, the 943rd Hospital of Joint Logistics Support Force of People's Liberation Army, Wuwei, China; ^5^ Department of Orthopaedics, No.905 Hospital of People's Liberation Army Navy, Second Military Medical University, Shanghai, China

**Keywords:** ewing sarcoma, multifocal metastases, multidisciplinary treatments, decision optimization, *en bloc* resection

## Abstract

Ewing sarcoma (ES) rarely derives from the sacrum or mobile spine. The discovery of primary ES with multimetastatic involvements is exceedingly less frequent in clinical practice. A 23-year-old man with initial primary sacral ES developed metastases of rib, lung, and multifocal skull after receiving surgical intervention and series of adjuvant therapies. We provide this very rare case consisting of its clinical features, imaging findings, treatments, and outcomes. Therapeutic modalities of ES are also reviewed in previous published articles. The prognosis of metastatic ES remains dismal; effective therapeutic modalities for ES require multidisciplinary collaboration, with more high-quality clinical trials to promote the optimal protocols.

## Introduction

Ewing sarcoma (ES) is a rare sarcoma with high aggressiveness and peak occurrence during 10–20 years old (1–3). ES generally originates from the diaphysis and metaphysis of long bones, pelvis, and ribs (4, 5) and relatively uncommonly in the spinal column (6), with fairly sparse cases of contemporary distal metastatic lesion involving the lung and skull. The common sites of metastasis are the lung and bone (7). Currently, the definitions of classic ES and peripheral primitive neuroectodermal tumors are overlapping (8) and uniformly classified as ES (9), having a similar histological appearance of uniform small round tumor cells and chromosome analysis of the most common t (10, 11)(q24; q12) translocation with functional fusion of the Ewing sarcoma breakpoint region 1 (*EWSR1*) gene and friend of leukemia virus integration site 1 gene (*FLI1*) (10, 12). ES in the sacrum and spine has worse prognosis than that in other sites (13). To the best of our knowledge, few studies reported the case of skull metastasis with or without systematic metastases (14–16). However, only one case originally arising from the sacrum was reported to develop lung and skull metastasis (17). Optimal favorable therapeutic protocols have not been established yet on such malignancy. Thus, we provide this very rare case including its clinical features, imaging findings, treatments, and outcomes. Therapeutic modalities of ES are also reviewed in previous published articles.

## Case presentation

**Figure 1:**
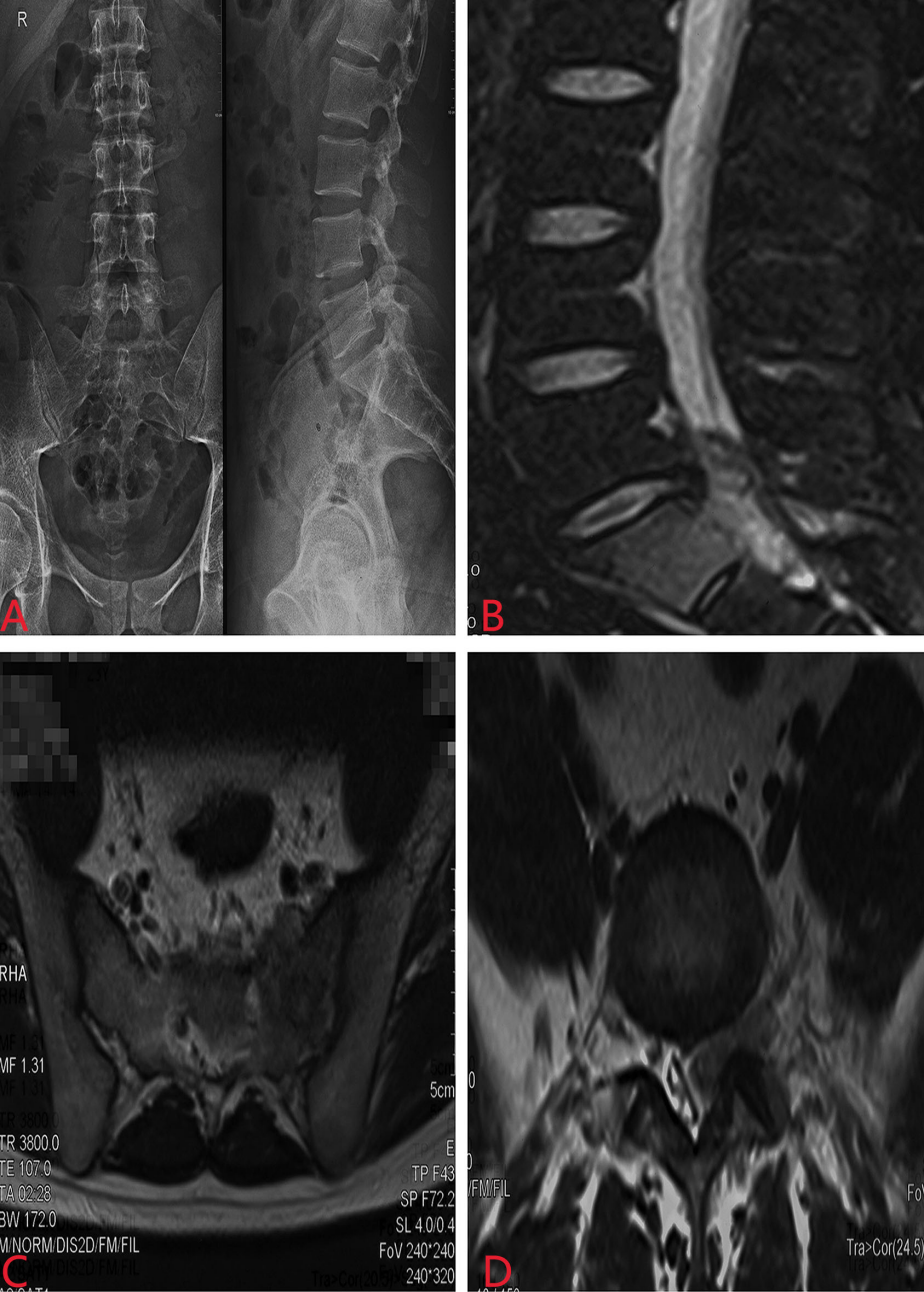
Preoperative X-ray and MRI revealed a space-occupying lesion with abnormal signal of the sacrum at the S1 level. **(A)** X-ray of the lumbosacral spine; **(B)** sagittal MRI; **(C)** coronal MRI; **(D)** lateral MRI.

A 23-year-old man developed initial back pain and progressively worsened in the following 6 months. It started to radiate to the hip with additional plantar numbness for 2 weeks; he came to the local hospital for medication and suggested to our department without regular treatments. However, he complained that his sleeping quality was extremely poor due to the night-increasing back pain, with weight loss of approximately 5 kg in the last half year. Physical examinations showed claudication, tenderness of the spinous process and perispinous process at L5–S1 level, paresthesia of the left lower extremity, abnormal Achilles tendon reflex, and positive Lasègue sign of the left lower limb. Magnetic resonance imaging (MRI) revealed a space-occupying lesion with abnormal signal of the sacrum at the S1 level (**Figure 1**). Routine blood tests revealed leukocytes as high as 17.5 * 10^9^/L (normal range 4–10 * 10^9^/L), with neutrophil–granulocyte ratio of 75.8%.

Full evaluation and written informed consent informing about the operation, as well as risks, were obtained after conducting necessary preoperative examinations. Intraoperative biopsy aiming at rapid diagnosis suggested the typical oncologic histology of uniform small round cells, so we removed the whole mass *via* an *en bloc* method, then bilateral vertebral pedicles at L4–L5 level and posterior superior iliac spine were used to accomplish spinal reconstruction. Emphasis was put on exposing the tumor margin to ensure a radical surgical procedure with a negative tumor margin. Oxaliplatin was used intraoperatively (50 ml:500 ml normal saline) for local chemotherapy on the basis of unbroken dura. The whole procedure lasted 3.5 h, with blood loss of about 1,200 ml. The postoperative physical examinations showed certain improvements of the motor and sensory functions 10 days after surgery. Subsequently, the hematoxylin–eosin (HE) and immunohistochemical staining indicated small round tumor cells with *CD99*(+), and histopathology confirmed the diagnosis of ES with molecular translocation t (10, 11)(q24;q12) with *EWSR1-FLI1* gene being identified. Postoperatively, he was transferred to the Tumor Hospital of Jiangxi Province, where he underwent two courses of external beam radiotherapy (EBRT) and four cycles of systemic chemotherapy. He received EBRT at a total dosage of 60 Gy with daily doses of 600 cGy (10 fractions in a month) and 30 Gy with daily doses of 200 cGy (15 times in a month). The chemotherapeutic protocol was cyclophosphamide, dactinomycin, vincristine, and doxorubicin (CAVD), and the blood test showed the quantity of leukocytes ranging from 2.32 to 2.55 * 10^9^/L. Good local control, satisfactory spinal reconstruction in the sacrum, and general condition of the lung without detecting metastasis were gained through physical and radiological examinations at 1, 6, and 12 months after surgery, respectively.

**Figure 2:**
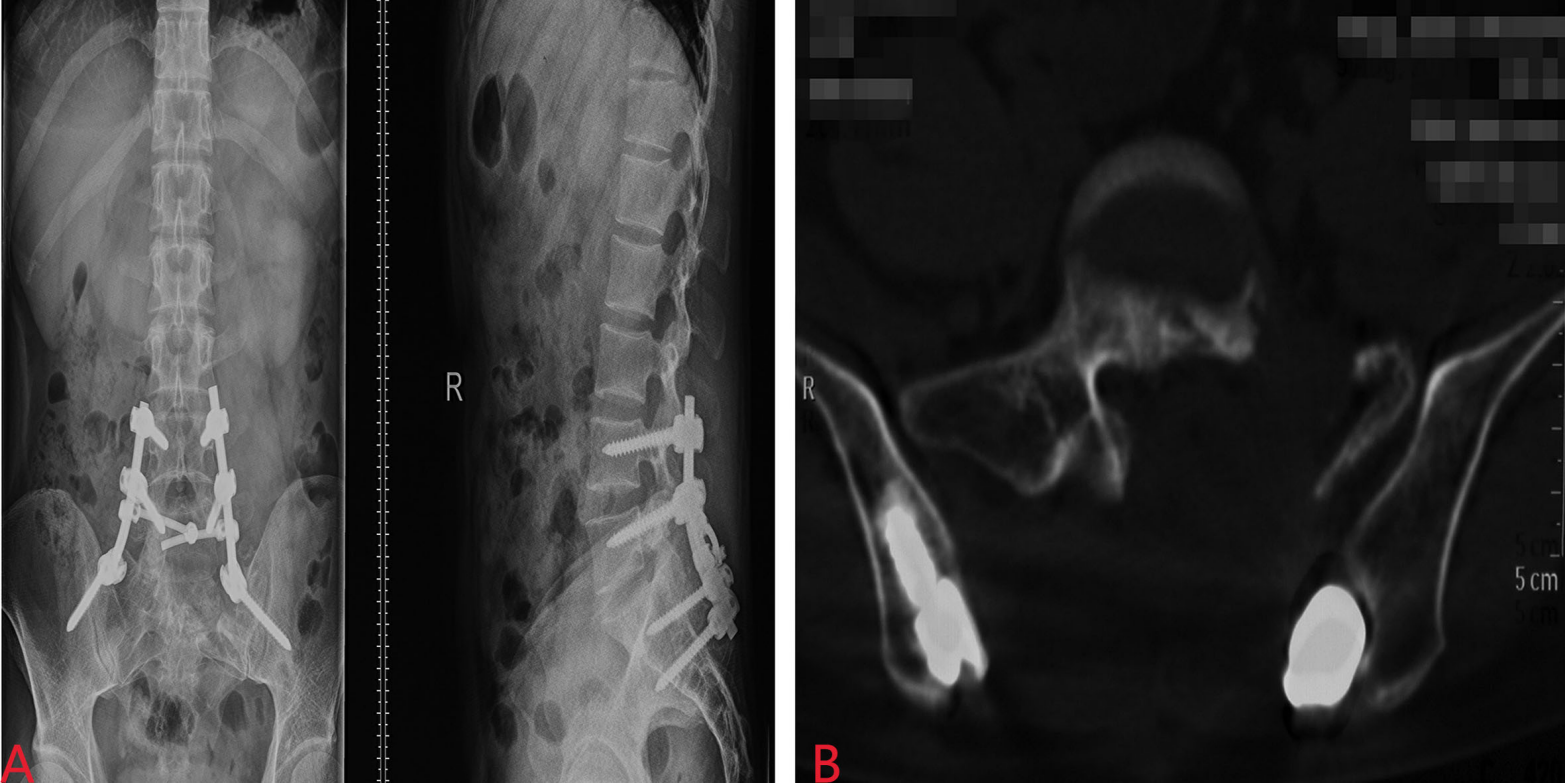
Postoperative X-ray **(A)** and CT **(B)** images did not indicate any change of the operative region.

**Figure 3:**
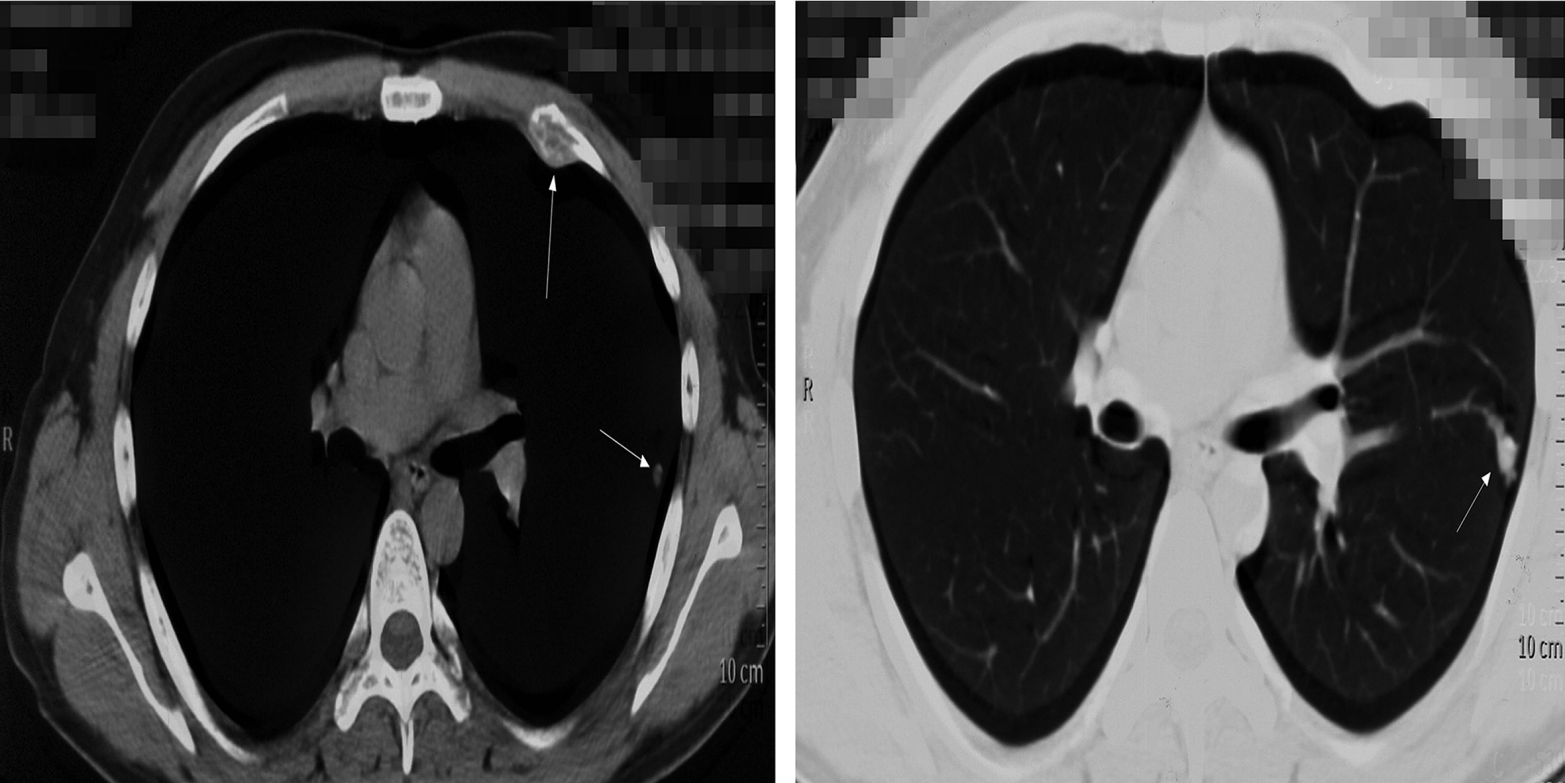
Thoracic CT demonstrated the metastatic lesions of the rib, left lung lobe. White arrows indicate the lesions.

**Figure 4:**
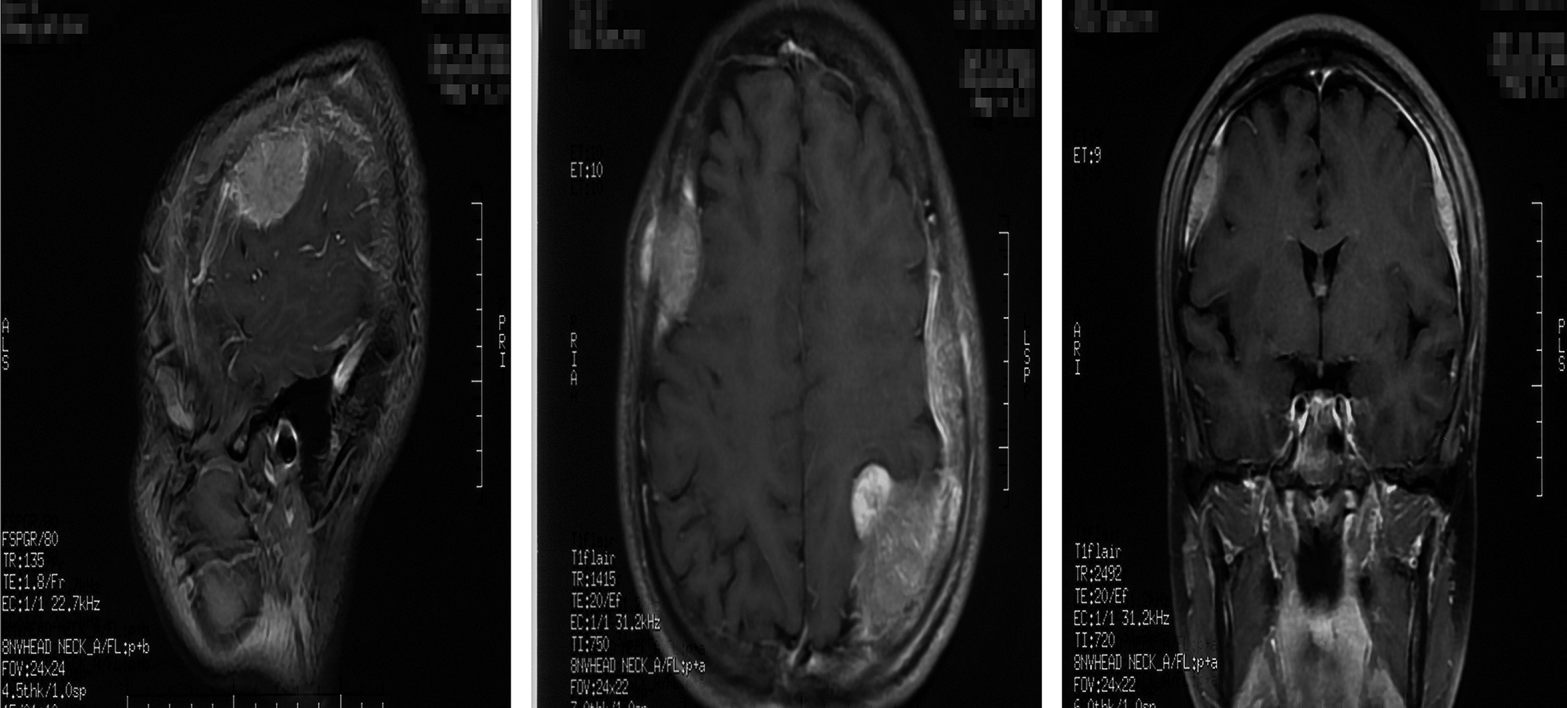
MRI of the brain showed the metastases of the scalp, skull, and meninges.

**Figure 5:**
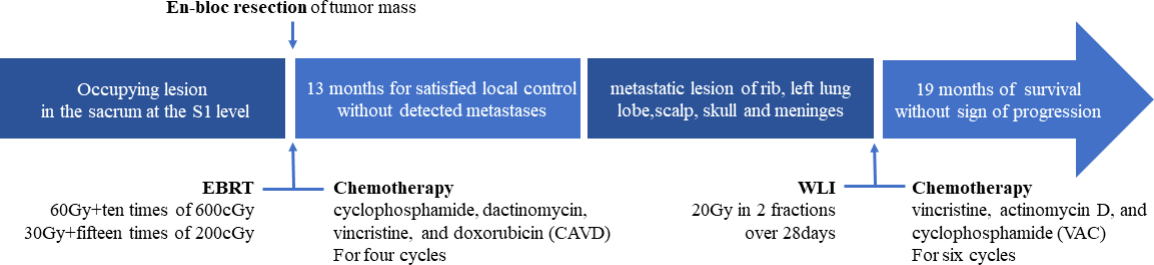
Timeline of patients undergoing multidisciplinary management.

However, the patient had been experiencing increasing headache 13 months after sacral ES excision. Although X-ray and computed tomography (CT) images did not indicate any change of the operative region (**Figure 2**), the thoracic CT and MRI of the brain demonstrated metastatic lesions of the rib, left lung lobe, scalp, skull, and meninges (**Figures 3**, **4**). Hence, besides changes in the chemotherapeutic program [vincristine, actinomycin D, and cyclophosphamide (VAC)] for six cycles, 20 Gy of whole-lung irradiation (WLI) was administered to him over 28 days in two fractions. His general condition and quality of daily life were temporarily stable without any sign of progressions at the final follow-up of 17 months. The timeline is shown in **Figure 5**.

## Discussion

### Epidemiology and clinical features

ES is the second most common primary malignancy of bone and soft tissue in adolescents and young adults after osteosarcoma, with an annual incidence of 1–3 persons per million and the highest morbidity during 10–20 years old (3, 18, 19). The incidence of ES among people of African and Asian ancestry is exceedingly rare (20). Both bone and soft tissue can be the primary lesions of ES with a relative incidence ratio of 7:3–17:3, and adults are prone to have extraosseous ES than children (21, 22). ES of the bone occurs mostly in the lower extremities and pelvis (1, 10), while the involvement of the spinal column only accounts for 3%–10% of all ESs (6, 22).

The common clinical features include local symptoms of mass formation, induration, pain, pathological fracture (11), and systemic characteristics of fever, anemia, fatigue, etc. (23). The typical radiographic finding is persistent osteolytic lesion with onion skin-like multilayered periosteal reaction and large soft tissue mass (24). The diagnostic histological appearance of ES is consistently small round and undifferentiated tumor cells with little cytoplasm (11) and frequent expressions of *CD99* (surface antigen *MIC2*) (25). Moreover, common translocation of the *EWSR1* gene from chromosome 22 to chromosome 11 is detected in 85% of cases (2, 26), producing the fusion protein product of *EWS-FLI1* (12).

The spinal column as the primary site of ES is rare; data derived from the Japanese Orthopaedic Association indicated that 23 of 326 ESs (7%) between 2006 and 2011 arose from the spine (11). In another study, Choi et al. (27) reported 13 patients who were diagnosed as having ES family tumors (ESFTs) in the spinal region that accounted for 14.3% of 91 cases during the period of July 1988 and July 2012. Another large-sample study demonstrated that 125 of 1,277 cases (9.8%) originated from the vertebral column (6); meanwhile, the sacrum as the primary lesion was detected in 67 cases (5.2%).

The distal metastatic rate can be 20%–30% of all cases, while most of the metastases are to the lung and/or bone (7, 28). Cranial with contemporary pulmonary metastasis from a primary lesion of the sacrum is extremely rare. To our knowledge, only one case originating from the sacrum had ever been reported to develop multiple frontal lobes as well as lung metastasis (17). Turgut et al. (29) reported a 22-year-old male patient with initial primary ES from the sacroiliac joint developing brain metastasis of the temporal lobe and parietal after 24-month treatments. As was reviewed, it was estimated that bone and soft tissue sarcomas accounted for only 0.8% of all cancers presenting with brain metastasis (30). Another study demonstrated that brain metastasis from ES merely made up <1.8% of all cases, with direct extension to adjacent central neural system rates up to 32%–56% (31). Notably, *via* reverse transcriptase polymerase chain reaction (RT-PCR), 20% of patients with a diagnosis of localized ES were detected to have widespread malignant tumor cells in the bone marrow and blood (32), of whom could be presumed to have subclinical metastatic disease.

### Multidisciplinary treatments and prognosis

Currently, the overall survival rate of patients with localized disease had been improved due to the evolutionary multidisciplinary management that includes local treatments and systematic treatments, with 5-year survival rates up to around 70% (10, 33). Initially, chemotherapy is effective against tumors, but relapses are common both locally and distantly (1). Moreover, patients with ESFTs deriving from the mobile spine and sacrum had an inferior 5-year survival rate of 42% (13). Metastatic status was found the strongest predictor of prognosis, with a 5-year survival rate of 20%–30% reported in previous articles (34, 35). What was worse was that patients with multifocal lesions tended to have a lower survival rate of <20% (36, 37). Disseminated primary disease and relapse accompanied by extremely poor outcomes urgently demand novel multidisciplinary treatments.

### Radiotherapy

For localized ES, surgical intervention and/or radiotherapy was recommended as an optimal modality for local control. Irradiation had been the first-line therapeutic modality since ESs are sensitive to radiotherapy (38, 39). With doses ranging from 36 to 60 Gy (40, 41), radiotherapy was adopted more frequently on cases concerning spinal column involvements, considering the difficulties of surgical resection and reconstruction of central locations (42, 43). However, definitive radiotherapy resulted in unfavorable prognosis with local recurrence rates of 33%–35%, but no significant difference seen in disease-free survival and overall survival, compared to surgeries (44, 45). In contrast, opposite results were observed on another retrospective study (46).

Moreover, radiation-induced second malignancy and adverse effects on growth especially for adolescents could be overlooked (47, 48). Indeed, radiotherapy is recommended as the first consideration only for inoperable lesions, with dosage ranging from 54 to 55 Gy (49). Additionally, it is recommended as an alternative option for intralesional or questionable surgical margins (34, 50).

### Surgical intervention

Surgical interventions on localized ES played a significant role in diagnosis, local control, and reconstruction of motor function (24). Current studies demonstrated that surgical modality might present more benefits than definitive radiotherapy, especially with wide or radical margins (27, 44, 51). From our perspectives, surgical interventions were capable of directly relieving tumor-related pain for primary spinal lesions and were able to immediately release the possible existing spinal cord compression to regain ambulatory status to obtain higher local control rates and enhance the quality of life. The specific surgical modality was associated with the patient’s general condition, the tumor location, the possibility for entire excision, and the patient’s willingness (52). As was acknowledged, the initial tumor volume was found robustly correlated with prognosis; a maximal diameter >8 cm (53) or a volume >200 ml (54) was regarded with poor survival. In addition, for resected ES, histological response was revealed as the strongest prognostic predictor rather than the tumor volume (54). However, direct comparison and further randomized trials between surgical interventions and radiotherapy were actually infeasible because of the patient selection bias. What could be recommended was that the combination of surgery and irradiation was definitely superior to definitive radiotherapy (27), with a 5-year survival rate of 72% compared to 27% in the previous study (55). Notably, preoperative and postoperative radiotherapy should be performed in cases of inadequate surgical margin (11).

### Systemic chemotherapy

For metastatic and localized ES, systemic chemotherapy remains a significant treatment modality. The relevant therapeutic drugs included vincristine (V), ifosfamide (I), dactinomycin (A), etoposide (E), cyclophosphamide (C), and doxorubicin (D), with the recommended combination of VIDE/VAI in Europe and VDC/IE in the United States (34, 36, 56). A randomized comparison between VAI and VAC elaborated that cyclophosphamide may be able to replace ifosfamide in the treatment of standard-risk ESs, which were defined as localized ESs with either a good histological response to chemotherapy (<10% viable cells) or small tumors (<200 ml) resected at diagnosis or receiving radiotherapy alone as local treatment (56). In addition, Womer et al. (57) demonstrated that the 5-year event-free survival (EFS) rate of patients with localized ES who were assigned to 2-week interval of receiving VDC-IE chemotherapy was higher than that of those assigned to 3-week treatment interval (73% vs. 65%, p = 0.048). Additionally, phased results of the Euro-Ewing 2012 trial show improved overall survival without an increase in toxicity for the VDC-IC group (58).

However, outcomes of systemic chemotherapy toward metastatic ES and recurrent and primary refractory Ewing sarcoma (rEECur) remained extremely poor, where 5-year OS rates are less than 30% (7) and 15% (59). Previous study revealed that the combination of IE and VACD did not improve outcomes with an 8-year EFS and OS of 20% and 32%, respectively. In addition, no significance was found between high-dose chemotherapy plus stem cell transplantation and conventional chemotharpy (60, 61). Notably, better prognosis was confirmed in patients with isolated pulmonary metastasis than those with bone/bone marrow and multisite metastases (7, 62). In the first randomized trial of rEECur, topotecan/cyclophosphamide (TC) or high-dose ifosfamide (IFOS) was demonstrated to be promising and the trial continues to recruit participants (59). Optimal therapeutic protocols on bone/bone marrow or multimetastatic patients remained a challenge for clinicians. The management of patients with metastases, recurrences, and weak responses requires robust evidence from multicenter trials conducted internationally.

Currently, chemotherapy was administered not only for metastatic control or prevention but also for local control as a neoadjuvant and/or postoperative modality (34). The current treatment algorithm includes neoadjuvant multiagent chemotherapy followed by local treatment, which can be either surgery or radiotherapy or a combination of both. We believed that preoperative chemotherapy should be applied in order to eliminate the potential micrometastasis and create opportunities for subsequent tumor resection *via* shrinking the tumor volume.

### Targeted therapy

Since the prognosis of patients with metastatic EFSTs remains dismal, molecular targets have been applied in the preclinical and clinical treatment protocols. Because of the difficulty of targeting fusion oncoproteins mediated by chimeric transcription factors, attention was shifted to alternative mechanisms regulated by oncogenic fusion transcription factors.

TK216 was designed to bind specifically to oncoproteins in the family of ETS transcription factors. In a phase I/II study, an overall clinical benefit rate of 64% (7/11) was observed after receiving TK216 alone or with vincristine (63). Insulin-like growth factor 1 receptor (*IGF-1R*) is one of the most important targets because tumor growth, metastasis, and angiogenesis are achieved through the activated *IGF-1R* pathway (26, 64). However, the application of *IGF-1R*-directed antibodies or small-molecule inhibitors was only able to provide a transient response in a low proportion of patients in several phase I/II clinical trials (65–67). *EWS-FLT1*, expressed in most ES cells, contains a DNA-binding domain at the C-terminus and could be an optimal target for new drugs at different expression levels (10). YK-4-279, known as a typical small-molecule inhibitor of RNA helicase A (*RHA*), has the capacity to interfere with the binding between *EWS-FLT1* and *RHA* to induce apoptosis in *in vitro* and *in vivo* experiments (68–70). Poly (adenosine diphosphate–ribose) polymerase (*PARP*) inhibitors are capable of interfering with the DNA repair process of ES in preclinical models, but they do not yield any positive results in clinical trials (71, 72). In addition, combination between targeted therapy and other therapeutic modalities may be more effective than using either alone (73). Nonetheless, although large quantities of targeted drugs had been invented and tested with definitive favorable results, more rigorous multicenter and large-scale clinical trials are required to detect the long-term effects and biological safety of molecular targeted drugs.

## Conclusions

In conclusion, effective treatment modalities for localized and metastatic ES require multidisciplinary collaboration, with more high-quality clinical trials to promote optimal therapeutic protocols.

## Data availability statement

The original contributions presented in the study are included in the article/supplementary material. Further inquiries can be directed to the corresponding authors.

## Ethics statement

Written informed consent was obtained from the individual(s) for the publication of any potentially identifiable images or data included in this article.

## Author contributions

SH and BX contributed to the implement of the treatment. WW, FL, and QC contributed to the collection and preparation of clinical data and graphic presentation, CY drafted the manuscript. JX and SH supervised and reviewed the writing. All authors approved the submitted version.

## Funding

This work was supported by a grant form the National Natural Science Foundation of China (82072971, Haifeng Wei). The funding source had no role in the study design, data gathering analysis, and interpretation, writing of the report, or the decision to submit the report for publication.

## Acknowledgments

The authors thank all the colleagues for great supports to this study and conscientious guidance.

## Conflict of interest

The authors declare that the research was conducted in the absence of any commercial or financial relationships that could be construed as a potential conflict of interest.

## Publisher’s note

All claims expressed in this article are solely those of the authors and do not necessarily represent those of their affiliated organizations, or those of the publisher, the editors and the reviewers. Any product that may be evaluated in this article, or claim that may be made by its manufacturer, is not guaranteed or endorsed by the publisher.
